# Association analysis of maternal MTHFR gene polymorphisms and the occurrence of congenital heart disease in offspring

**DOI:** 10.1186/s12872-021-02117-z

**Published:** 2021-06-14

**Authors:** Mengting Sun, Tingting Wang, Peng Huang, Jingyi Diao, Senmao Zhang, Jinqi Li, Liu Luo, Yihuan Li, Letao Chen, Yiping Liu, Jianhui Wei, Xinli Song, Xiaoqi Sheng, Jiabi Qin

**Affiliations:** 1 Department of Epidemiology and Health Statistics, Xiangya School of Public Health, Central South University, 110 Xiangya Road, Changsha, 410078 Hunan China; 2 NHC Key Laboratory of Birth Defect for Research and Prevention, Hunan Provincial Maternal and Child Health Care Hospital, 78 Xiangchun Road, Changsha, 410008 Hunan China; 3 Department of Cardiothoracic Surgery, Hunan Children’s Hospital, Changsha, Hunan China; 4 Guangdong Cardiovascular Institute, Guangdong Provincial People’s Hospital, Guangdong Academy of Medical Sciences, Guangzhou, Guangdong China; 5 Hunan Provincial Key Laboratory of Clinical Epidemiology, Hunan, China

**Keywords:** Congenital heart disease, Folate metabolism, MTHFR, Polymorphisms, Interaction

## Abstract

**Background:**

Although many studies showed that the risk of congenital heart disease (CHD) was closely related to genetic factors, the exact pathogenesis is still unknown. Our study aimed to comprehensively assess the association of single nucleotide polymorphisms (SNPs) of maternal MTHFR gene with risk of CHD and its three subtypes in offspring.

**Methods:**

A case–control study involving 569 mothers of CHD cases and 652 health controls was conducted. Thirteen SNPs were detected and analyzed.

**Results:**

Our study showed that genetic polymorphisms of maternal MTHFR gene at rs4846052 and rs1801131 were significantly associated with risk of CHD in the homozygote comparisons (TT vs. CC at rs4846052: OR = 7.62 [95%CI 2.95–19.65]; GG vs. TT at rs1801131: OR = 5.18 [95%CI 2.77–9.71]). And six haplotypes of G–C (involving rs4846048 and rs2274976), A–C (involving rs1801133 and rs4846052), G–T (involving rs1801133 and rs4846052), G–T–G (involving rs2066470, rs3737964 and rs535107), A–C–G (involving rs2066470, rs3737964 and rs535107) and G–C–G (involving rs2066470, rs3737964 and rs535107) were identified to be significantly associated with risk of CHD. Additionally, we observed that a two-locus model involving rs2066470 and rs1801131 as well as a three-locus model involving rs227497, rs1801133 and rs1801131 were significantly associated with risk of CHD in the gene–gene interaction analyses. For three subtypes including atrial septal defect, ventricular septal defect and patent ductus arteriosus, similar results were observed.

**Conclusions:**

Our study indicated genetic polymorphisms of maternal MTHFR gene were significantly associated with risk of fetal CHD in the Chinese population. Additionally, there were significantly interactions among different SNPs on risk of CHD. However, how these SNPs affect the development of fetal heart remains unknown, and more studies in different ethnic populations and with a larger sample are required to confirm these findings.

**Supplementary Information:**

The online version contains supplementary material available at 10.1186/s12872-021-02117-z.

## Introduction

Congenital heart disease (CHD) is the most common birth defect, with an estimated prevalence rate of 8.6–10.3 per 1000 live births and rising [1, 2]. Although the ability of diagnosis and treatment of CHD has been greatly improved in recent decades, it is undeniable that the disease still causes a considerable disease burden in many countries. And the cost and complexity of treating CHD is much higher than many other children's diseases [3]. In view of China's huge population base and unreasonable distribution of health resources, CHD is also a serious problem threatening the health of children. In China, the incidence of CHD has reached 8.98 per 1000 live births [4, 5].

In order to reduce the impact of CHD on human health, many researchers have devoted themselves to the etiology of CHD. Presently, it is widely believed that CHD is a multifactorial disease involving environmental and genetic factors [6–8]. Some epidemiological evidence showed that one-third of CHD cases can be explained by genetic factors [9]. Additionally, the etiology of CHD involves the gene–gene and gene-environment interactions, indicating its complexity [10–12].

It has been reported that single nucleotide polymorphisms (SNP) of some genes, such as Notch1, GATA4, NKX2-5,TBX5 and so on, were significantly associated with the risk of CHD [13, 14]. Among these genes related to CHD, 5,10-methylenetetrahydrofolate reductase (MTHFR) gene is one of the most popular candidate genes. The MTHFR gene also plays an important role in the formation of many other congenital defects, such as neural tube defects, and cleft lip and palate [15, 16]. In 2001, researchers proposed the association between MTHFR genetic polymorphisms and risk of CHD for the first time [17]. The possible mechanism is related to folic acid and homocysteine metabolism. Folic acid deficiency can lead to hyperhomocysteinemia, which has been recognized as a risk factor of CHD [18]. The MTHFR gene is one of the most critical genes in the process of folate/homocysteine metabolism. The MTHFR encoded by the MTHFR gene is involved in the process of homocysteine metabolism, catalyzing the conversion of 5,10-methylenetetrahydrofolate into 5-methylenetetrahydrofolate. The methyl produced can make homocysteine produce methionine and avoid the accumulation of homocysteine in the body due to abnormal metabolism [19, 20]. Therefore, the genetic variants of maternal MTHFR gene may alter the susceptibility to CHD in offspring by influencing the folate/homocysteine metabolism.

Presently, although many studies have assessed the association of MTHFR genetic polymorphisms with the risk of CHD, these studies only focused on two loci including rs1801133 and rs1801131, and the results were often inconsistent [8, 19, 21]. Actually, there are still many other functional loci for MTHFR gene, which have not received enough attentions in the field of CHD. The present study aimed to further assess the association of 13 SNPs (i.e.,* rs2274976, rs1801133, rs535107, rs4846052, rs1476413, rs4846048, rs4846051, rs1931226, rs2066470, rs3737964, rs7525338, rs1801131 and rs1889292*) of maternal MTHFR gene with risk of CHD and its three subtypes in offspring.

## Methods

### Ethical statement

This study was complied with the Declaration of Helsinki and approved by the Ethics Committee of Xiangya School of Public Health, Central South University (No. XYGW-2018-36). The protocol of this study was registered at the Chinese Clinical Trial Registry with registration number ChiCTR1800016635 and is available at http://www.chictr.org.cn/listbycreater.aspx. All participants provided written informed consent before completing an enrollment questionnaire as well as providing biological samples.

### Study population

In this study, a hospital-based case–control design was adopted. All subjects were recruited from Hunan children’s Hospital from November 2017 to January 2020. Mothers of CHD children under one year old who visited the Department of cardiothoracic surgery of Hunan children's Hospital were recruited into the case group. Mothers of healthy children under one year old who visited the Department of Child Healthcare during the same period were recruited into the control group after health counselling or a medical examination.

### Inclusion and exclusion criteria

In the present study, the exposures of interest were genetic polymorphisms of maternal MTHFR gene. The outcomes of interest were CHD including the following subtypes: atrial septal defect (ASD), ventricular septal defect (VSD), patent ductus arteriosus (PDA), aorto-pulmonary window (APW), tetralogy of Fallot (TOF) and complete transposition of great arteries (TGA). The diagnosis of CHD was confirmed by echocardiography and/or surgery. All eligible mothers belonged to singleton pregnancies for this pregnancy, were of Han Chinese descent, had a complete record of questionnaire, and provided the blood sample. We only concerned non-syndromic CHD, and patients with structural malformations involving another organ system or known chromosomal abnormalities were excluded. Participants who reported a history of depression or other psychiatric disorders or were diagnosed with depression or a psychiatric illness were also excluded when they were recruited into the study.

### Information collection

In order to fully understand the situation of all subjects, and then control the possible confounding factors in the later analysis, we specially designed a questionnaire for this research. The face-to-face interview was adopted to complete the questionnaire by professionally trained investigators. The questionnaire used has been described in our previous published articles [22]. Here we collected maternal social demographic characteristics (e.g.,* age, education level, family income in the past 1 year, body mass index before this pregnancy, and residence location*), abnormal pregnancy history before this pregnancy (e.g.,* spontaneous abortion, induced abortion or labor, fetal death or stillbirth, premature delivery, low birth weight, neonatal death, ectopic pregnancy, hypertension of pregnancy, and gestational diabetes mellitus*), family history (e.g.,* consanguineous marriages and congenital malformations*), personal lifestyle and habit before this pregnancy in the 3 months before this pregnancy (e.g.,* active smoking, passive smoking, drinking, and drinking tea*), exposure history of environmental hazardous substance in the 3 months before this pregnancy (e.g.,* harmful chemicals, noise pollution exposure, newly renovated houses, dyeing or perming hair, and frequency of cosmetics use*) and medicine history in this pregnancy (e.g.,* folate use, macrolide antibiotics, and antidepressants*).

After completing the questionnaire, all mothers were requested to provide 3–5 ml of peripheral venous blood for genotyping. Blood samples were collected in EDTA-treated (ethylenediamine tetraacetic acid) anticoagulant tubes and then immediately centrifuged into plasma and blood cells. Blood cells were separated and stored at − 80 °C until genotyping was performed. Genomic DNA was extracted from peripheral venous blood sample using the QIAamp DNA Mini Kit (Qiagen, Valencia, CA, USA) according to the manufacturer's standard protocol and dissolved in sterile TBE buffer.

### SNPs selection and genotyping

The selection of candidate loci of MTHFR gene has been described by a published study [23]. Briefly, SNP markers were selected using the SNPBrowser™ program (version 3.0) provided by AppliedBiosystems Inc. This program allowed selection of SNP markers from the HapMap database (http://www.hapmap.org/). For each target gene, tagging SNPs were selected based on the pairwise r^2^ ≥ 0.8. These SNPs with minor allele frequencies lower than 10% were excluded. As a result, these genetic loci (*rs2274976, rs1801133, rs535107, rs4846052, rs1476413, rs4846048, rs4846051, rs1931226, rs2066470, rs3737964, rs7525338, rs1801131 and rs1889292*) of MTHFR gene, were selected as candidate loci for this study. The polymorphisms of MTHFR gene were genotyped by the matrix-assisted laser desorption and ionization time-of-flight mass spectrometry Mass Array system (Agena iPLEXassay, San Diego, CA, USA). The error rate for genotyping was less than 5%. The experimenters who performed the genotyping were not informed in advance of the status of the control or case groups. Each sample was retyped and double-checked to ensure the reliability of the experiments.

### Statistical analysis

Categorical variables were presented in absolute numbers or as percentages. Continuous variables were described using means and standard deviations (SD). Differences of unordered categorical variables between two groups were calculated by Chi-square test or Fisher's exact test. Wilcoxon rank sum test was used to compare the difference in ordinal categorical variables and Student-t-test was for numerical variables. The Hardy–Weinberg equilibrium (HWE) was additionally tested in control group (significance level at *P* < 0.05). False discovery rate *P* value (FDR_*P*), which was adjusted for multiple testing, was estimated to get a more precise *P* value. All odds ratio (OR) and 95% confidence intervals (CIs) were calculated by logistic regression analysis to show the level of association. Multivariate logistic regression analysis was used to adjust for the baseline data with statistical significance in Table 1 to further assess the association of maternal MTHFR gene polymorphisms with the risk of CHD in offspring. In the present study, we comprehensively analyzed the association of genotype and three genetic models (i.e.,* dominant model, recessive model and additive model*) for every SNP with the risk of CHD. Linkage disequilibrium test (whether there was a strong association between the two SNPs was judged by r^2^ ≥ 0.8) and haplotype analysis were adopted to analyze the association between each haplotype and the risk of CHD. The interaction effects of different SNPs on the development of CHD were evaluated by generalized multifactor dimensionality reduction (GMDR) method (using GMDR 0.9 software). The accuracy and CV Consistency of each model in training samples and test samples were calculated, and the statistical significance of each model was determined by symbolic test. The FDR_*P* values were calculated by using R software (version 4.0.2, *SNPassoc* package). Linkage disequilibrium test and haplotype analysis was performed in Haploview 4.2 software. Other analyses were performed using SAS 9.1 (SAS Institute, Cary, NC, USA). Significance was set at a *P* value less than 0.05 (two-tailed). Of note, we focused not only on the risk of total CHD, but also on the risk of three CHD subtypes including ASD, VSD and PDA. However, when assessing the interaction effects of different SNPs, we only examined the risk of total CHD instead of the risk of specific subtypes due to the limited sample size.

**Table 1:** Comparison of maternal baseline characteristics in cases and controls

Variables	Control group (n = 652)	Case group (n = 569)	Univariable analysis
*Demographic characteristics*			
Maternal age (years)	28.60 ± 4.75	28.23 ± 5.07	
< 35	560 (85.9%)	494 (86.8%)	*χ*^2^ = 0.222*; P* = 0.637
≥ 35	92 (14.1%)	75 (13.2%)	
Education level			
Less than primary or primary	9 (1.4%)	85 (14.9%)	Z = -14.298; *P* = 0.000^*^
Junior high school	127 (19.5%)	231 (40.6%)	
Senior middle school	210 (32.2%)	162 (28.5%)	
College and above	306 (46.9%)	91 (16.0%)	
Family income in the past 1 year (RMB)			
≤ 50,000	187 (28.7%)	463 (81.4%)	Z = -18.157; *P* = 0.000^*^
50,000–100,000	275 (42.2%)	77 (13.5%)	
100,000–150,000	59 (9.0%)	11 (1.9%)	
> 150,000	131 (20.1%)	18 (3.2%)	
Body mass index before this pregnancy			
< 18.5	149 (22.8%)	125 (22.0%)	*χ*^2^ = 0.204*; P* = 0.903
18.5–23.99	391 (60.0%)	342 (60.1%)	
≥ 24	112 (17.2%)	102 (17.9%)	
Residence location (rural areas)	349 (53.5%)	428 (75.2%)	*χ*^2^ = 61.784*; P* = 0.000
*Abnormal pregnancy history before this pregnancy*			
Spontaneous abortion (yes)	55 (8.4%)	68 (12.0%)	*χ*^2^ = 4.144*; P* = 0.042
Induced abortion or labor (yes)	208 (31.9%)	242 (42.5%)	*χ*^2^ = 14.750*; P* = 0.000
Fetal death or stillbirth (yes)	2 (0.3%)	19 (3.3%)	*χ*^2^ = 16.530*; P* = 0.000
Premature delivery (yes)	4 (0.6%)	8 (1.4%)	*χ*^2^ = 1.961*; P* = 0.161
Low birth weight (yes)	3 (0.5%)	5 (0.9%)	*χ*^2^ = 0.818*; P* = 0.366
Neonatal death (yes)	0	7 (1.2%)	*P* = 0.005^**^
Ectopic pregnancy (yes)	18 (2.8%)	12 (2.1%)	*χ*^2^ = 0.539*; P* = 0.463
Hypertension of pregnancy (yes)	12 (1.8%)	39 (6.9%)	*χ*^2^ = 19.082*; P* = 0.000
Gestational diabetes mellitus (yes)	23 (3.5%)	59 (10.4%)	*χ*^2^ = 22.701*; P* = 0.000
*Family history*			
Consanguineous marriages (yes)	2 (0.3%)	24 (4.2%)	*χ*^2^ = 22.302*; P* = 0.000
Congenital malformations (yes)	4 (0.6%)	40 (7.0%)	*χ*^2^ = 36.010*; P* = 0.000
*Personal lifestyle and habit before this pregnancy in the 3 months before this pregnancy*			
Active smoking (yes)	12 (1.8%)	46 (8.1%)	*χ*^2^ = 26.180*; P* = 0.000
Passive smoking (yes)	249 (38.2%)	293 (51.5%)	*χ*^2^ = 21.785*; P* = 0.000
Drinking (yes)	45 (6.9%)	78 (13.7%)	*χ*^2^ = 15.538*; P* = 0.000
Drinking tea (yes)	128 (19.6%)	77 (13.5%)	*χ*^2^ = 8.091*; P* = 0.004
*Exposure history of environmental hazardous substance in the 3 months before this pregnancy*			
Harmful chemicals (yes)	42 (6.4%)	119 (20.9%)	*χ*^2^ = 55.592*; P* = 0.000
Noise pollution exposure (yes)	121 (18.6%)	152 (26.7%)	*χ*^2^ = 11.641*; P* = 0.000
Newly renovated houses (yes)	37 (5.7%)	44 (7.7%)	*χ*^2^ = 2.078*; P* = 0.149
Dyeing or perming hair (yes)	42 (6.4%)	70 (12.3%)	*χ*^2^ = 12.526*; P* = 0.000
Frequency of cosmetics use			
Never	409 (62.7%)	418 (73.5%)	Z = -3.106; *P* = 0.002^*^
Sometime	160 (24.5%)	66 (11.6%)	
Often	37 (5.7%)	36 (6.3%)	
Every day	46 (7.1%)	49 (8.6%)	
*Medicine history in this pregnancy*			
Folate use (no)	44 (6.7%)	95 (16.7%)	*χ*^2^ = 29.803*; P* = 0.000
Macrolide antibiotics (yes)	23 (3.6%)	35 (6.2%)	*χ*^2^ = 4.622*; P* = 0.032
Antidepressants (yes)	2 (0.3%)	10 (1.8%)	*χ*^2^ = 6.571*; P* = 0.010

^*^Differences between cases and controls were tested by Wilcoxon rank-sum test

^**^Differences between cases and controls were tested by Fisher’s exact test

## Results

### Characteristics of study participants

In this study, total 569 eligible mothers were recruited into the case group, 652 into the control group. Among 569 CHD cases, 95 were diagnosed with ASD, 353 with VSD, 170 with PDA, 32 with TOF, 8 with APW, and 2 with TGA. Of note, some cases have been diagnosed with multiple subtypes of CHD. Therefore, the sum of the various subtypes was not equal to 569. Statistically significant differences were observed between two groups for maternal education level, family income, residence location, abnormal pregnancy history, family consanguineous marriage history, family congenital malformation history, personal lifestyle and habit before pregnancy, exposure history to environmentally hazardous substances as well as medicine history in this pregnancy (Table 1). These factors were adjusted when assessing the association of maternal MTHFR gene polymorphisms and their interactions with the risk of CHD in offspring.

### Association of maternal MTHFR gene polymorphisms with the risk of CHD in offspring

Maternal MTHFR genotype frequencies and *P* values of HWE test are summarized in Table 2. The genotype distributions in the control group conformed to HWE for all SNPs. However, only a few variant genotypes were observed for three loci including rs4846051, rs1931226 and rs7525338. Therefore, we did not further analyze them.

**Table 2:** Maternal MTHFR genotype frequencies and *P* values of HWE test

SNPs	Chromosome	Major allele	Minor allele	MAF	group	Genotype frequencies^†^			HWE test *P*
						AA	AB	BB	
rs2274976	1:11790870	C	T	0.1183	Control	505 (77.5%)	136 (20.9%)	11 (1.7%)	0.5995
					Case	448 (78.7%)	111 (19.5%)	10 (1.8%)	
rs1801133	1:11796321	G	A	0.3141	Control	308 (47.2%)	289 (44.3%)	55 (8.4%)	0.2651
					Case	245 (43.1%)	280 (49.2%)	44 (7.7%)	
rs535107	1:11829411	A	G	0.2404	Control	402 (61.7%)	216 (33.1%)	34 (5.2%)	0.4798
					Case	314 (55.2%)	207 (36.4%)	48 (8.4%)	
rs4846052	1:11797894	C	T	0.1057	Control	567 (87.0%)	79 (12.1%)	6 (0.9%)	0.0884
					Case	437 (76.8%)	97 (17.0%)	35 (6.2%)	
rs1476413	1:11792243	C	T	0.2391	Control	399 (61.2%)	217 (33.3%)	36 (5.5%)	0.3668
					Case	330 (58.0%)	183 (32.2%)	56 (9.8%)	
rs4846048	1:11786195	A	G	0.1212	Control	522 (80.1%)	119 (18.3%)	11 (1.7%)	0.1703
					Case	433 (76.1%)	117 (20.6%)	19 (3.3%)	
rs4846051	1:11794400	A	G	0.0020	Control	649 (99.5%)	3 (0.5%)	0	0.9530
					Case	567 (99.6%)	2 (0.4%)	0	
rs1931226	1:11803917	C	T	0.0012	Control	649 (99.5%)	3 (0.5%)	0	0.9530
					Case	569 (100.0%)	0	0	
rs2066470	1:11803000	G	A	0.1167	Control	517 (79.3%)	126 (19.3%)	9 (1.4%)	0.0606
					Case	443 (77.9%)	111 (19.5%)	15 (2.6%)	
rs3737964	1:11806987	C	T	0.1200	Control	523 (80.2%)	118 (18.1%)	11 (1.7%)	0.1544
					Case	430 (75.6%)	125 (22.0%)	14 (2.5%)	
rs7525338	1:11802275	C	T	0.0020	Control	649 (99.5%)	3 (0.5%)	0	0.9530
					Case	567 (99.6%)	2 (0.4%)	0	
rs1801131	1:11794419	T	G	0.2138	Control	448 (68.7%)	188 (28.8%)	16 (2.5%)	0.4750
					Case	323 (56.8%)	190 (33.4%)	56 (9.8%)	
rs1889292	1:11780886	C	T	0.2445	Control	377 (57.8%)	246 (37.7%)	29 (4.4%)	0.1586
					Case	318 (55.9%)	209 (36.7%)	42 (7.4%)	

*MTHFR* Methylenetetraphydrofolate reductase, *SNP* single nucleotide polymorphism, *HWE* Hardy–Weinberg equilibrium, *MAF* minimum allele frequency

^†^AA = homozygous wild-type; AB = heterozygous variant type; BB = homozygous variant type

Associations of maternal MTHFR genetic polymorphisms with risks of total CHD and its three subtypes in offspring based on multivariate logistic regression analysis are summarized in Table 3. After adjustment for potential confounding factors, our study showed that the genetic polymorphism of maternal MTHFR gene at rs4846052 was significantly associated with the risk of total CHD (TT vs. CC: OR = 7.62 [95%CI 2.95–19.65]; dominant model: OR = 1.97 [95%CI 1.41–2.75]; recessive model: OR = 7.12 [95%CI 2.76–18.33]; additive model: OR = 1.96 [95%CI 1.48–2.60]) and its three subtypes including ASD (TT vs. CC: OR = 7.56 [95%CI 2.39–23.90]; recessive model: OR = 7.04 [95%CI 2.23–22.16]; additive model: OR = 2.14 [95%CI 1.39–3.28]), VSD (TT vs. CC: OR = 5.12 [95%CI 1.92–13.65]; recessive model: OR = 4.93 [95%CI 1.85–13.13]; additive model: OR = 1.62 [95%CI 1.20–2.19]), and PDA (TT vs. CC: OR = 6.57 [95%CI 2.34–18.44]; recessive model: OR = 6.63 [95%CI 2.37–18.54]; additive model: OR = 1.61 [95%CI 1.12–2.32]) (all FDR_*P* values ≤ 0.05).

**Table 3:** Associations of maternal MTHFR gene polymorphisms with risk of CHD based on multivariate logistic regression analysis

SNPs	Total CHD^†^			ASD^†^			VSD^†^			PDA^†^		
	OR_adj_ (95%CI)	*P*	FDR_*P*	OR_adj_ (95%CI)	*P*	FDR_*P*	OR_adj_ (95%CI)	*P*	FDR_*P*	OR_adj_ (95%CI)	*P*	FDR_*P*
*rs2274976*												
CC	1			1			1			1		
CT	0.83 (0.61–1.14)	0.254	0.462	0.98 (0.57–1.67)	0.928	0.999	0.79 (0.56–1.12)	0.184	0.409	0.53 (0.31–0.89)	0.017	0.113
TT	1.01 (0.36–2.80)	0.987	0.987	-	0.999	0.999	1.39 (0.50–3.86)	0.534	0.763	1.26 (0.39–4.12)	0.699	0.873
Dominant model	0.84 (0.62–1.15)	0.278	0.397	0.92 (0.54–1.58)	0.768	0.913	0.83 (0.59–1.16)	0.267	0.445	0.59 (0.37–0.96)	0.033	0.083
Recessive model	1.05 (0.38–2.91)	0.925	0.925	-	0.999	0.999	1.45 (0.52–4.03)	0.473	0.676	1.37 (0.42–4.48)	0.606	0.673
Additive model	0.87 (0.66–1.15)	0.341	0.426	0.88 (0.53–1.45)	0.606	0.895	0.89 (0.66–1.20)	0.428	0.653	0.70 (0.46–1.06)	0.090	0.180
*rs1801133*												
GG	1			1			1			1		
GA	1.28 (0.98–1.66)	0.072	0.240	1.46 (0.91–2.35)	0.120	0.400	1.35 (1.01–1.81)	0.041	0.205	1.52 (1.03–2.24)	0.034	0.125
AA	1.22 (0.76–1.97)	0.410	0.547	1.75 (0.79–3.86)	0.166	0.463	1.05 (0.62–1.78)	0.852	0.949	0.89 (0.38–2.10)	0.786	0.873
Dominant model	1.27 (0.98–1.63)	0.069	0.230	1.50 (0.95–2.37)	0.079	0.225	1.30 (0.99–1.72)	0.064	0.213	1.44 (0.98–2.11)	0.060	0.120
Recessive model	1.08 (0.68–1.70)	0.742	0.824	1.45 (0.69–3.08)	0.329	0.823	0.90 (0.55–1.49)	0.687	0.859	0.71 (0.31–1.62)	0.412	0.515
Additive model	1.17 (0.96–1.43)	0.115	0.192	1.37 (0.97–1.93)	0.072	0.240	1.15 (0.93–1.43)	0.201	0.503	1.21 (0.89–1.63)	0.228	0.285
*rs535107*												
AA	1			1			1			1		
AG	1.16 (0.88–1.52)	0.285	0.407	1.53 (0.95–2.44)	0.078	0.400	1.03 (0.76–1.39)	0.854	0.949	1.15 (0.78–1.70)	0.472	0.640
GG	1.55 (0.93–2.59)	0.096	0.274	1.23 (0.48–3.15)	0.672	0.999	1.64 (0.96–2.80)	0.073	0.243	1.54 (0.78–3.05)	0.213	0.387
Dominant model	1.22 (0.94–1.57)	0.135	0.270	1.48 (0.94–2.33)	0.090	0.225	1.12 (0.85–1.48)	0.437	0.568	1.22 (0.84–1.75)	0.298	0.373
Recessive model	1.47 (0.89–2.43)	0.138	0.230	1.02 (0.41–2.56)	0.967	0.999	1.62 (0.96–2.74)	0.073	0.168	1.46 (0.75–2.83)	0.265	0.515
Additive model	1.20 (0.98–1.48)	0.074	0.247	1.29 (0.90–1.84)	0.166	0.415	1.16 (0.93–1.45)	0.178	0.503	1.20 (0.91–1.60)	0.201	0.285
*rs4846052*												
CC	1			1			1			1		
CT	1.56 (1.09–2.24)	0.015	0.100	1.64 (0.89–2.99)	0.110	0.400	1.28 (0.86–1.90)	0.219	0.438	0.94 (0.54–1.64)	0.831	0.875
TT	7.62 (2.95–19.65)	0.000	0.000	7.56 (2.39–23.90)	0.001	0.020	5.12 (1.92–13.65)	0.001	0.010	6.57 (2.34–18.44)	0.000	0.000
Dominant model	1.97 (1.41–2.75)	0.000	0.000	2.14 (1.25–3.66)	0.006	0.060	1.56 (1.09–2.24)	0.016	0.080	1.40 (0.87–2.24)	0.165	0.236
Recessive model	7.12 (2.76–18.33)	0.000	0.000	7.04 (2.23–22.16)	0.001	0.010	4.93 (1.85–13.13)	0.001	0.005	6.63 (2.37–18.54)	0.000	0.000
Additive model	1.96 (1.48–2.60)	0.000	0.000	2.14 (1.39–3.28)	0.001	0.010	1.62 (1.20–2.19)	0.002	0.010	1.61 (1.12–2.32)	0.011	0.050
*rs1476413*												
CC	1			1			1			1		
CT	0.97 (0.74–1.28)	0.819	0.862	1.25 (0.79–1.98)	0.349	0.635	0.71 (0.52–0.97)	0.031	0.205	0.86 (0.58–1.30)	0.480	0.640
TT	1.50 (0.92–2.45)	0.107	0.268	0.37 (0.09–1.61)	0.185	0.463	1.62 (0.97–2.72)	0.065	0.243	1.72 (0.92–3.21)	0.091	0.184
Dominant model	1.05 (0.81–1.36)	0.704	0.880	1.12 (0.71–1.76)	0.623	0.890	0.85 (0.64–1.13)	0.255	0.445	1.01 (0.69–1.46)	0.978	0.978
Recessive model	1.52 (0.94–2.46)	0.091	0.227	0.34 (0.08–1.45)	0.144	0.480	1.82 (1.09–3.01)	0.021	0.070	1.81 (0.98–3.32)	0.057	0.173
Additive model	1.11 (0.91–1.35)	0.314	0.449	0.97 (0.66–1.41)	0.862	0.895	1.01 (0.81–1.26)	0.910	0.910	1.13 (0.85–1.49)	0.399	0.443
*rs4846048*												
AA	1			1			1			1		
AG	1.09 (0.79–1.50)	0.622	0.732	1.14 (0.67–1.94)	0.635	0.999	1.13 (0.80–1.60)	0.484	0.755	1.45 (0.94–2.24)	0.092	0.184
GG	2.50 (1.11–5.63)	0.027	0.135	-	0.999	0.999	0.99 (0.37–2.69)	0.991	0.992	2.64 (1.00–6.95)	0.050	0.125
Dominant model	1.20 (0.89–1.63)	0.239	0.398	1.04 (0.61–1.77)	0.878	0.913	1.12 (0.80–1.56)	0.511	0.568	1.57 (1.05–2.37)	0.029	0.083
Recessive model	2.46 (1.09–5.54)	0.030	0.100	-	0.999	0.999	0.97 (0.36–2.63)	0.956	0.956	2.44 (0.93–6.38)	0.069	0.173
Additive model	1.26 (0.97–1.63)	0.085	0.170	0.95 (0.58–1.55)	0.829	0.895	1.09 (0.81–1.45)	0.575	0.653	1.53 (1.09–2.14)	0.015	0.050
*rs2066470*												
GG	1			1			1			1		
GA	0.90 (0.65–1.24)	0.505	0.631	1.35 (0.81–2.27)	0.251	0.502	0.92 (0.64–1.30)	0.628	0.794	0.60 (0.36–0.99)	0.048	0.125
AA	1.74 (0.65–4.66)	0.272	0.418	-	0.999	0.999	2.26 (0.87–5.86)	0.093	0.266	1.21 (0.34–4.27)	0.767	0.873
Dominant model	0.95 (0.69–1.29)	0.727	0.808	1.25 (0.75–2.09)	0.395	0.688	1.00 (0.72–1.40)	0.995	0.995	0.65 (0.40–1.04)	0.075	0.125
Recessive model	1.78 (0.67–4.75)	0.252	0.360	-	0.999	0.999	2.31 (0.89–5.96)	0.084	0.168	1.29 (0.37–4.57)	0.690	0.690
Additive model	1.00 (0.76–1.32)	0.983	0.983	1.12 (0.70–1.80)	0.643	0.895	1.08 (0.81–1.45)	0.588	0.653	0.74 (0.49–1.12)	0.150	0.250
*rs3737964*												
CC	1			1			1			1		
CT	1.26 (0.92–1.73)	0.154	0.308	1.37 (0.81–2.34)	0.242	0.502	1.13 (0.80–1.61)	0.481	0.755	1.63 (1.05–2.54)	0.030	0.125
TT	1.66 (0.69–3.96)	0.257	0.428	-	0.999	0.999	1.00 (0.37–2.69)	0.992	0.992	1.74 (0.61–4.99)	0.303	0.505
Dominant model	1.30 (0.95–1.76)	0.097	0.243	1.25 (0.74–2.11)	0.413	0.688	1.12 (0.80–1.57)	0.509	0.568	1.65 (1.08–2.50)	0.020	0.083
Recessive model	1.58 (0.66–3.78)	0.300	0.375	-	0.999	0.999	0.97 (0.36–2.63)	0.956	0.956	1.58 (0.55–4.50)	0.396	0.515
Additive model	1.27 (0.97–1.65)	0.079	0.198	1.10 (0.68–1.77)	0.707	0.895	1.09 (0.81–1.46)	0.575	0.653	1.50 (1.05–2.13)	0.025	0.063
*rs1801131*												
TT	1			1			1			1		
TG	1.30 (0.98–1.71)	0.067	0.268	1.48 (0.92–2.39)	0.105	0.400	1.12 (0.82–1.52)	0.491	0.755	1.48 (1.00–2.18)	0.050	0.125
GG	5.18 (2.77–9.71)	0.000	0.000	4.55 (1.67–12.34)	0.003	0.030	4.98 (2.66–9.33)	0.000	0.000	3.65 (1.63–8.18)	0.002	0.020
Dominant model	1.58 (1.22–2.06)	0.001	0.005	1.68 (1.07–2.65)	0.025	0.125	1.43 (1.08–1.90)	0.014	0.080	1.66 (1.14–2.40)	0.008	0.080
Recessive model	4.76 (2.56–8.85)	0.000	0.000	3.99 (1.49–10.71)	0.006	0.030	4.82 (2.59–8.98)	0.000	0.000	3.18 (1.44–7.02)	0.004	0.020
Additive model	1.68 (1.36–2.09)	0.000	0.000	1.75 (1.19–2.56)	0.004	0.020	1.59 (1.27–1.99)	0.000	0.000	1.67 (1.23–2.26)	0.001	0.010
*rs1889292*												
CC	1			1			1			1		
CT	0.96 (0.73–1.25)	0.742	0.824	0.98 (0.62–1.56)	0.933	0.999	0.80 (0.60–1.08)	0.139	0.348	0.98 (0.67–1.43)	0.903	0.903
TT	1.49 (0.86–2.59)	0.152	0.338	0.93 (0.31–2.82)	0.902	0.999	1.16 (0.64–2.11)	0.635	0.794	1.40 (0.67–2.93)	0.368	0.566
Dominant model	1.02 (0.79–1.32)	0.892	0.892	0.98 (0.62–1.53)	0.913	0.913	0.84 (0.64–1.12)	0.230	0.445	1.03 (0.71–1.48)	0.881	0.978
Recessive model	1.52 (0.89–2.60)	0.128	0.256	0.94 (0.32–2.80)	0.912	0.999	1.26 (0.70–2.27)	0.441	0.676	1.42 (0.69–2.91)	0.342	0.515
Additive model	1.08 (0.88–1.33)	0.477	0.530	0.98 (0.66–1.43)	0.895	0.895	0.92 (0.73–1.16)	0.490	0.653	1.08 (0.80–1.45)	0.619	0.619

*MTHFR* Methylenetetraphydrofolate reductase, *CHD* congenital heart disease, *ASD* atrial septal defect, *VSD* ventricular septal defect, *PDA* patent ductus arteriosus, *SNP* single nucleotide polymorphism, *OR*_*adj*_ adjusted odds ratio, *CI* confidence interval, *FDR* false discovery rate

^†^Adjusted for maternal education, family income, residence location, abnormal pregnancy history, family consanguineous marriage history, family congenital malformation history, personal lifestyle and habit before pregnancy, exposure history to environmentally hazardous substances as well as medicine history in this pregnancy

Additionally, the genetic polymorphism of maternal MTHFR gene at rs1801131 was also significantly associated with the risk of total CHD (GG vs. TT: OR = 5.18 [95%CI 2.77–9.71]; dominant model: OR = 1.58 [95%CI 1.22–2.06]; recessive model: OR = 4.76 [95%CI 2.56–8.85]; additive model: OR = 1.68 [95%CI 1.36–2.09]) and its three subtypes including ASD (GG vs. TT: OR = 4.55 [95%CI 1.67–12.34]; recessive model: OR = 3.99 [95%CI 1.49–10.71]; additive model: OR = 1.75 [95%CI 1.19–2.56]), VSD (GG vs. TT: OR = 4.98 [95%CI 2.66–9.33]; recessive model: OR = 4.82 [95%CI 2.59–8.98]; additive model: OR = 1.59 [95%CI 1.27–1.99]), and PDA (GG vs. TT: OR = 3.65 [95%CI 1.63–8.18]; recessive model: OR = 3.18 [95%CI 1.44–7.02]; additive model: OR = 1.67 [95%CI 1.23–2.26]) (all FDR_*P* values ≤ 0.05).

### Linkage disequilibrium test and haplotype analysis

**Fig. 1:**
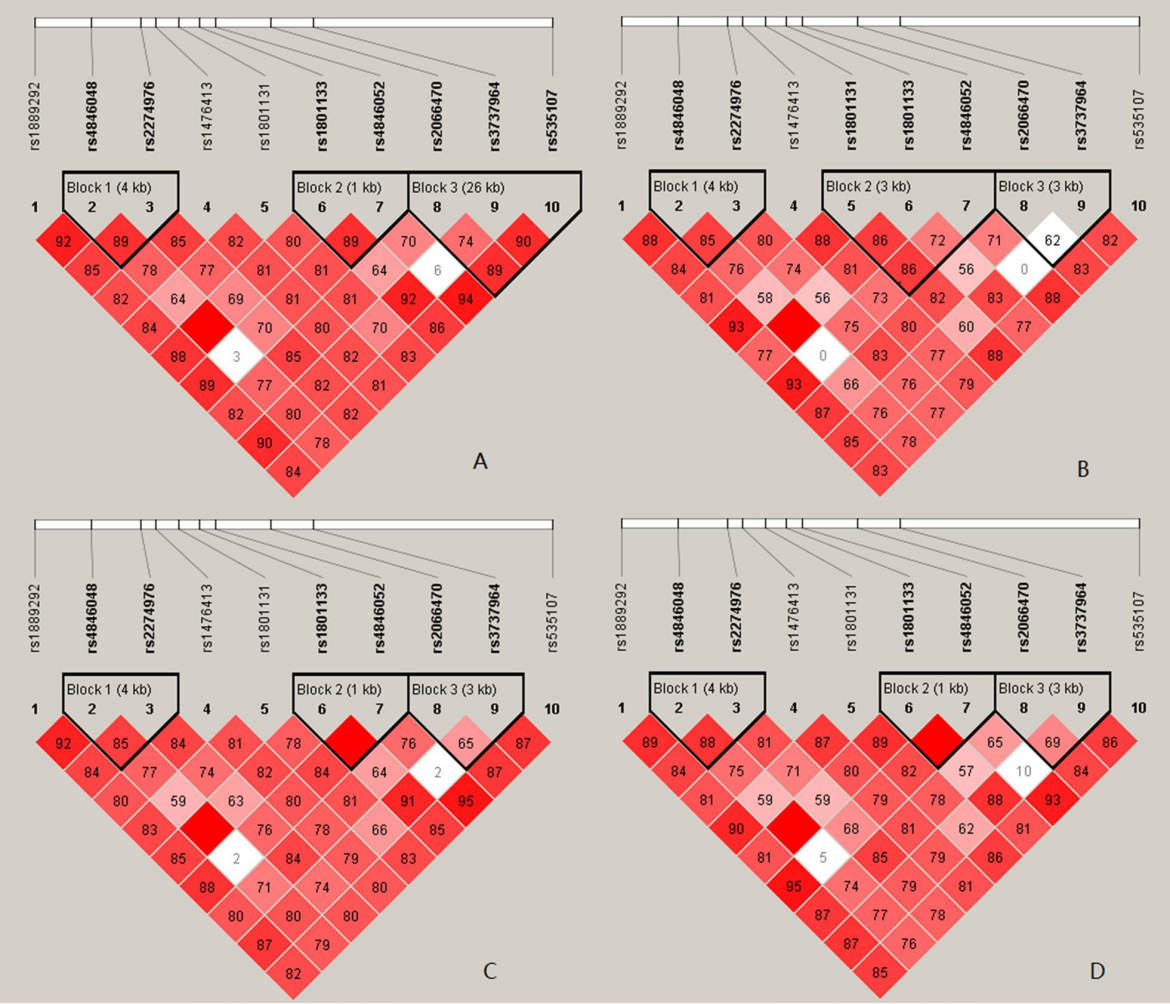
Linkage disequilibrium tests for maternal MTHFR genetic polymorphisms across different comparison groups (A: total CHD group vs. controls; B: ASD group vs. controls; C: VSD group vs. controls; D: PDA group vs. controls)

The r-square values of linkage disequilibrium test for maternal MTHFR genetic polymorphisms are summarized in Additional file 1: Table S1; Additional file 2: Table S2; Additional file 3: Table S3; Additional file 4: Table S4. Our results showed that there were not strong correlations between 10 SNPs for different comparison groups (all r^2^ values < 0.8). As shown in Fig. 1, the r^2^ values and log-odds scores indicated that these SNPs constructed three potential linkage disequilibrium blocks across different comparison groups. The haplotype frequencies of maternal MTHFR genetic polymorphisms across different comparison groups are summarized in Table 4. For the risk of total CHD, six haplotypes of G–C (involving rs4846048 and rs2274976; OR = 1.31 [95%CI 1.02–1.67]), A–C (involving rs1801133 and rs4846052; OR = 1.22 [95%CI 1.02–1.46]), G–T (involving rs1801133 and rs4846052; OR = 2.43 [95%CI 1.84–3.21]), G–T–G (involving rs2066470, rs3737964 and rs535107; OR = 1.60 [95%CI 1.23–2.08]), A–C–G (involving rs2066470, rs3737964 and rs535107; OR = 1.39 [95%CI 1.07–1.81]) and G–C–G (involving rs2066470, rs3737964 and rs535107; OR = 0.29 [95%CI 0.15–0.55]) were identified.

**Table 4:** Haplotype frequencies of maternal MTHFR genetic polymorphisms across different comparison groups

Haplotypes	Controls (%)	Cases (%)	OR (95% CI)^†^	*P*
*Total CHD group versus the control group*				
rs4846048-rs2274976				
A–C	1007.3 (77.2)	853.5 (75.0)	1	
G–C	138.7 (10.6)	153.5 (13.5)	1.31 (1.02–1.67)	0.034
A–T	155.7 (11.9)	129.5 (11.4)	0.98 (0.76–1.26)	0.884
rs1801133-rs4846052				
G–C	816.3 (62.6)	609.3 (53.5)	1	
A–C	396.7 (30.4)	361.7 (31.8)	1.22 (1.02–1.46)	0.027
G–T	88.7 (6.8)	160.7 (14.1)	2.43 (1.84–3.21)	< 0.001
rs2066470-rs3737964-rs535107				
G–C–A	979.8 (75.1)	832.5 (73.2)	1	
G–T–G	112.2 (8.6)	152.6 (13.4)	1.60 (1.23–2.08)	< 0.001
A–C–G	117.0 (9.0)	138.6 (12.2)	1.39 (1.07–1.81)	0.013
G–C–G	47.5 (3.6)	11.6 (1.0)	0.29 (0.15–0.55)	< 0.001
*The ASD group versus the control group*				
rs4846048-rs2274976				
A–C	1007.4 (77.3)	146.4 (77.1)	1	
A–T	155.6 (11.9)	20.6 (10.8)	0.91 (0.56–1.49)	0.710
G–C	138.6 (10.6)	22.6 (11.9)	1.12 (0.70–1.81)	0.636
rs1801131-rs1801133-rs4846052				
T–G–C	687.7 (52.7)	73.1 (38.5)	1	
T–A–C	388.1 (29.8)	59.7 (31.4)	1.45 (1.01–2.08)	0.046
G–G–C	137.2 (10.5)	26.2 (13.8)	1.80 (1.11–2.91)	0.017
G–G–T	74.5 (5.7)	22.1 (11.6)	2.79 (1.64–4.75)	< 0.001
rs2066470-rs3737964				
G–C	1025.9 (78.7)	142.0 (74.7)	1	
A–C	138.1 (10.6)	24.0 (12.7)	1.26 (0.79–2.00)	0.340
G–T	134.1 (10.3)	23.0 (12.1)	1.24 (0.77–1.99)	0.377
*The VSD group versus the control group*				
rs4846048-rs2274976				
A–C	1007.4 (77.3)	535.5 (75.0)	1	
A–T	155.6 (11.9)	81.5 (13.5)	0.99 (0.74–1.31)	0.920
G–C	138.6 (10.6)	87.5 (11.4)	1.19 (0.89–1.58)	0.241
rs1801133-rs4846052				
G–C	814.0 (62.4)	378.0 (53.5)	1	
A–C	399.0 (30.6)	232.0 (31.8)	1.25 (1.02–1.53)	0.030
G–T	91.0 (7.0)	96.0 (14.1)	2.27 (1.66–3.10)	< 0.001
rs2066470-rs3737964				
G–C	1025.8 (78.7)	530.4 (73.2)	1	
A–C	138.2 (10.6)	87.6 (12.2)	1.23 (0.92–1.63)	0.165
G–T	134.2 (10.3)	84.6 (1.0)	1.22 (0.91–1.63)	0.183
*The PDA group versus the control group*				
rs4846048-rs2274976				
A–C	1007.3 (77.2)	246.4 (72.5)	1	
G–C	138.7 (10.6)	62.6 (18.4)	1.85 (1.33–2.57)	< 0.001
A–T	155.7 (11.9)	30.6 (9.0)	0.80 (0.53–1.21)	0.298
rs1801133-rs4846052				
G–C	814.0 (62.4)	186.0 (54.7)	1	
A–C	399.0 (30.6)	103.0 (30.3)	1.13 (0.86–1.48)	0.374
G–T	91.0 (7.0)	51.0 (15.0)	2.45 (1.68–3.58)	< 0.001
rs2066470-rs3737964				
G–C	1025.6 (78.7)	245.0 (72.1)	1	
G–T	134.4 (10.3)	62.0 (18.2)	1.93 (1.39–2.69)	< 0.001
A–C	138.4 (10.6)	32.0 (9.4)	0.97 (0.64–1.46)	0.876

*MTHFR* Methylenetetraphydrofolate reductase, *CHD* congenital heart disease, *ASD* atrial septal defect, *VSD* ventricular septal defect, *PDA* patent ductus arteriosus, *OR* odds ratio, *CI* confidence interval

^†^The OR values and 95% CIs were calculated using binary logistic regression

For the risk of ASD, three haplotypes (involving rs1801131, rs1801133 and rs4846052) of T–A–C (OR = 1.45 [95%CI 1.01–2.08]), G–G–C (OR = 1.80 [95%CI 1.11–2.91]) and G–G–T (OR = 2.79 [95%CI 1.64–4.75]) were identified. For the risk of VSD, two haplotypes (involving rs1801133 and rs4846052) of A–C (OR = 1.25 [95%CI 1.02–1.53]) and G–T (OR = 2.27 [95%CI 1.66–3.10]) were identified. For the risk of PDA, three haplotypes of G–C (involving rs4846048 and rs2274976; OR = 1.85 [95%CI 1.33–2.57]), G–T (involving rs1801133 and rs4846052; OR = 2.45 [95%CI 1.68–3.58]) and G–T (involving rs2066470 and rs3737964; OR = 1.93 [95%CI 1.39–2.69]) were identified.

### Interactions between different SNPs on the risk of total CHD

In the present study, we assessed the associations of interactions between different SNPs with the risk of total CHD by using GMDR method (Table 5). As a result, a two-locus model involving rs2066470 and rs1801131 was identified to be significantly associated with the risk of total CHD (*P* = 0.0010), the cross-validation consistency of which was 10/10. Besides, a significant three-locus model involving rs227497, rs1801133 and rs1801131 was also identified (*P* = 0.0010), the cross-validation consistency of which was 5/10.

**Table 5:** Best gene–gene interaction models identified by GMDR

Interaction model	Training bal. acc	Testing bal. acc	*P*^†^	CV consistency
rs1801133	0.5487	0.5282	0.0547	9/10
rs2066470-rs1801131	0.5956	0.5929	0.0010	10/10
rs2274976-rs1801133-rs1801131	0.6232	0.5961	0.0010	5/10

*GMDR* generalized multifactor dimensionality reduction

^†^Adjusted for maternal education, family income, residence location, abnormal pregnancy history, family consanguineous marriage history, family congenital malformation history, personal lifestyle and habit before pregnancy, exposure history to environmentally hazardous substances as well as medicine history in this pregnancy

We further conducted a hierarchical analysis for the significant models identified in the GMDR analysis by using multivariate logistic regression analysis (Table 6). After adjustment for potential confounding factors, for two-locus model, our study showed mothers carrying rs2066470-GG and rs1801131-TG/GG genotype were at a significantly higher risk of total CHD in offspring compared with the reference group (OR = 2.16 [95%CI 1.41–3.30]). For three-locus model, the present study showed that mothers carrying rs2274976-CC, rs1801133-GG and rs1801131-TG/GG genotype (OR = 2.08 [95%CI 1.17–3.67]), rs2274976-CC, rs1801133-GA/AA and rs1801131-TT genotype (OR = 1.87 [95%CI 1.24–2.82]) as well as rs2274976-CC, rs1801133-GA/AA and rs1801131-TG/GG genotype (OR = 6.18 [95%CI 2.83–13.51]) had a significantly increased risk of total CHD compared with the reference group.

**Table 6:** Hierarchical analysis for gene–gene interactions by using logistic regression

Variable 1	Variable2	Variable 3	OR (95% CI)^†^	*P*
*rs2066470-rs1801131 interaction*				
rs2066470	rs1801131			
GG	TT		1	
GA + AA	TT		1.69 (0.51–5.53)	0.390
GG	TG + GG		2.16 (1.41–3.30)	0.000
GA + AA	TG + GG		0.87 (0.59–1.29)	0.496
*rs2274976-rs1801133-rs1801131 interaction*				
rs2274976	rs1801133	rs1801131		
CC	GG	TT	1	
CT + TT	GG	TT	–	–
CC	GG	TG + GG	2.08 (1.17–3.67)	0.012
CT + TT	GG	TG + GG	1.16 (0.68–2.01)	0.584
CC	GA + AA	TT	1.87 (1.24–2.82)	0.003
CT + TT	GA + AA	TT	0.91 (0.68–1.65)	0.511
CC	GA + AA	TG + GG	6.18 (2.83–13.51)	0.000
CT + TT	GA + AA	TG + GG	1.25 (0.69–2.28)	0.464

*OR* odds ratio, *CI* confidence interval

^†^Adjusted for maternal education, family income, residence location, abnormal pregnancy history, family consanguineous marriage history, family congenital malformation history, personal lifestyle and habit before pregnancy, exposure history to environmentally hazardous substances as well as medicine history in this pregnancy

## Discussion

In the present study, we assessed the association of 13 SNPs of maternal MTHFR gene with the risk of CHD in offspring. After adjustment for potential confounding factors, our study suggested that genetic polymorphisms of maternal MTHFR gene at rs4846052 and rs1801131 were significantly associated with the susceptibility of CHD in offspring. Our study also showed six haplotypes of G–C (involving rs4846048 and rs2274976), A–C (involving rs1801133 and rs4846052), G–T (involving rs1801133 and rs4846052), G–T–G (involving rs2066470, rs3737964 and rs535107), A–C-G (involving rs2066470, rs3737964 and rs535107) and G–C–G (involving rs2066470, rs3737964 and rs535107) were identified to be significantly associated with risk of CHD. Additionally, we observed that a two-locus model involving rs2066470 and rs1801131as well as a three-locus model involving rs227497, rs1801133 and rs1801131 were significantly associated with risk of CHD in the gene–gene interaction analyses. For three CHD subtypes including ASD, VSD and PDA, similar results were observed. These results highlight the important role of genetic elements in the development of CHD. Similarly, it reveals that maternal genetic factors can serve as potential biomarkers for CHD screening in offspring.

Folate deficiency plays an important role in the etiology of CHD [24]. Many studies have shown that the genetic variants of key enzyme genes in folate metabolism pathway, such as MTHFR, methionine synthase reductase (MTRR), methionine synthase (MTR) and cystathionine beta synthase (CBS) were significantly associated with the occurrence of CHD [22, 25, 26]. Here, we investigated the association of maternal MTHFR gene polymorphisms closely related to folate metabolism with the risk of CHD in offspring. Previous studies mainly focused on the genetic polymorphisms of MTHFR gene at rs1801133 and rs1801131. It has confirmed that genetic variants of these two SNPs were significantly associated with lower activity of MTHFR, which can further reduce the concentration of folate in plasma while increase the level of homocysteine [21, 27–29]. There were some studies showing genetic variant of rs1801133 in mothers was a risk factor for fetal CHD in the Chinese Han population [30, 31]. In other populations, the genetic variant of rs1801133 was also found to be significantly associated with the risk of fetal CHD [32, 33]. However, a meta-analysis suggested that genetic polymorphism of MTHFR gene at rs1801133 was not significantly associated with the risk of CHD [34]. Our study also did not find a significantly positive association between maternal genetic polymorphism of rs1801133 and CHD risk in offspring.

The present study also showed that genetic polymorphism of rs1801131 was significantly associated with the risk of fetal CHD. For example, mothers with the GG genotype compared with those with the TT genotype had a significantly higher risk of CHD in offspring (OR = 5.18), which was supported by previous studies [35]. So far, few studies have simultaneously detected multiple SNPs to comprehensively analyze the association of maternal MTHFR gene polymorphisms with the risk of fetal CHD. Therefore, a lot of important genetic information will inevitably be missed when assessing the association between MTHFR gene and CHD. In the present study, we focused not only on the above-mentioned two SNPs (i.e.,* rs1801133 and rs1801131*), but also on other 11 SNPs of MTHFR gene (i.e.,* rs2274976, rs535107, rs4846052, rs1476413, rs4846048, rs4846051, rs1931226, rs2066470, rs3737964, rs7525338 and rs1889292*). Here, we found the genetic variant of maternal MTHFR gene at rs4846052 was a risk factor for CHD in offspring. To our knowledge, this is the first time that the associations of maternal MTHFR gene polymorphisms with fetal CHD were comprehensively assessed, which could help to provide some new clues for screening candidate genes of CHD.

In addition, considering the possible interactions between different SNPs, we first analyzed the haplotypes of MTHFR gene and the interactions between MTHFR SNPs in CHD in order to avoid ignoring the real effect of genetic variation. Although in single locus analysis, we failed to find the association between rs1801133 of MTHFR gene in mothers and CHD in offspring. Interestingly, it was found that the haplotype block formed by rs1801133 and rs4846052 was associated with risk of CHD in our study. In the meantime, rs2274976, rs1801133 and rs1801131 formed a significant three locus model through GMDR software analysis of gene–gene interaction, indicating that the combination of these three SNPs in mothers was significantly associated with risk of fetal CHD. By analyzing the haplotypes and their interactions between different SNPs of MTHFR gene, we concluded that the risk of CHD in offspring is not only limited by the polymorphism of single SNP, but also by the interaction between SNPs. However, more researches are needed to demonstrate this view in the future.

Although the association between MTHFR gene and CHD has been extensively studied, the molecular mechanism of MTHFR gene and cardiac dysplasia remains unclear. The MTHFR gene is located on chromosome 1p36.3. When the SNPs of MTHFR gene mutated, it would lead to the decrease of MTHFR enzyme activity and the abnormality of folate metabolism, thus interfering with the development of cardiovascular and nervous system in embryo [36]. It was hypothesized that genetic polymorphisms in folate-metabolizing enzymes affect DNA methylation as well as changes in the availability of nucleotides for the synthesis and repair of DNA [37]. An animal experiment found that mice knocking out the MTHFR gene decreased DNA methylation ability [38]. Besides, DNA methylation was found in myocardial biopsy of patients with TOF and VSD [39]. All the above findings provided evidence for the close association between MTHFR gene and DNA methylation in CHD cases. In addition, folic acid was involved in the process of DNA synthesis/methylation, and the level of folic acid was directly related to DNA methylation [40]. Therefore, folate metabolism played an important role in the stability of genome.

MicroRNAs (miRNAs) are small endogenous non-encoding RNAs, which have about 22 nucleotides [41]. Previous studies have shown that miRNAs were an important presence in CHD, and miRNAs based drugs would bring new hope for the treatment of cardiovascular diseases [42–45]. It has been reported that folic acid deficiency and DNA hypomethylation can lead to misexpression of miRNAs [46]. A cancer study found that mir-22-3p inhibited MTHFR expression when folic acid was deficient [47]. The latest preliminary report pointed out that the genetic polymorphism of MTHFR gene at rs4846048increased the risk of cervical cancer through its association with miR-522 [48]. Interestingly, we found that the offspring of mothers carrying the haplotype G–C (involving rs4846048 and rs2274976) had an increased risk of CHD (OR = 1.31). Therefore, the appearance of miRNAs provides us with a new idea for studying the mechanism of folate deficiency and MTHFR gene polymorphisms leading to CHD. Can it be assumed that SNPs of MTHFR are associated with miRNAs involved in the formation of CHD? A similar point of view was also put forward in the study of neural tube defects [49].

Several limitations are relevant to our study. Firstly, because children with CHD are a relatively special population, it was impossible to select the study participants by random sampling. Therefore, it was likely to bring about selection bias. The convenience sample, driven mainly by the number of respondents, was used for our study. This limitation could lead to subsequent problems, including sample representativeness and generalization of study findings. Secondly, in this study, cases were recruited from the Department of cardiothoracic surgery, and controls were recruited from the Department of Child Healthcare. Because the cases and controls did not come from the same sample source, the balance of baseline characteristics between the two groups was affected. However, we adjusted the baseline characteristics when exploring the association between maternal MTHFR gene polymorphisms and the occurrence of CHD in offspring. Thirdly, although we adjusted for a large number of potential confounding factors, there was still no guarantee that the results would not be affected by potential residual confounding factors. Fourthly, owing to the limitation of sample size, we only assessed three CHD subtypes. Fifthly, in view of the obvious ethnic and regional differences in MTHFR gene polymorphisms [50], it is necessary to conduct this study in larger and different ethnic populations, and then compare the genetic susceptibility of different populations. Moreover, several key enzyme genes of folate metabolism pathway as an etiological factor of CHD have attracted extensive attention. However, we only considered the association between maternal MTHFR gene and fetal CHD in this study. In the future, we could assess the associations of SNPs of folate metabolism-related genes (i.e.,* MTHFR, MTRR and MTR*) and their interactions with the risk of CHD. These limitations highlight the urgent need for large samples and different ethnic populations to further confirm our findings.

## Conclusion

This is the first study to comprehensively assess the association of 13 SNPs of maternal MTHFR gene with the risk of CHD in offspring. The present findings indicate that genetic polymorphisms of maternal MTHFR gene at rs4846052 and rs1801131 are significantly associated with higher risk of CHD in offspring. Additionally, our study supports a significant association of six haplotypes of G–C (involving rs4846048 and rs2274976), A–C (involving rs1801133and rs4846052), G–T (involving rs1801133and rs4846052), G–T–G (involving rs2066470, rs3737964 and rs535107), A–C–G (involving rs2066470, rs3737964 and rs535107) and G–C-G (involving rs2066470, rs3737964 and rs535107) with risk of CHD. A significant two-locus model involving rs2066470 and rs1801131 as well as three-locus model involving rs227497, rs1801133 and rs1801131 were observed among gene–gene interaction analyses. However, how these SNPs affect the development of fetal heart remains unknown, and more studies in different ethnic populations and with a larger sample are required to confirm these findings.

## Supplementary Information

**Additional file 1: Table S1.** Degree of linkage disequilibrium of MTHFR genetic polymorphisms between total CHD group and control group.

**Additional file 2: Table S2.** Degree of linkage disequilibrium of MTHFR genetic polymorphisms between ASD group and control group.

**Additional file 3: Table S3.** Degree of linkage disequilibrium of MTHFR genetic polymorphisms between VSD group and control group.

**Additional file 4: Table S4.** Degree of linkage disequilibrium of MTHFR genetic polymorphisms between PDA group and control group.

## Data Availability

The datasets used and/or analyzed during the current study are available from the corresponding author on reasonable request.

## Abbreviations

MTHFR: 5,10-methylenetetrahydrofolate reductase
CHD: congenital heart disease
SNPs: single nucleotide polymorphisms
SD: standard deviations
HWE: the Hardy–Weinberg equilibrium
FDR_*P*: false discovery rate *P* value
OR: odds ratio
CIs: confidence intervals
GMDR: generalized multifactor dimensionality reduction
MTRR: methionine synthase reductase
MTR: methionine synthase
CBS: cystathionine beta synthase
ASD: atrial septal defect
VSD: ventricular septal defect
PDA: Patent ductus arteriosus
APW: Aorto-pulmonary window
TOF: tetralogy of Fallot
TGA: complete transposition of great arteries
miRNAs: microRNAs
