# Supplementary material for: Association analysis of maternal MTHFR gene polymorphisms and the occurrence of congenital heart disease in offspring
Source: BMC Cardiovasc Disord. 2021 Jun 14;21:298. doi: 10.1186/s12872-021-02117-z (PMC8204503; doi:10.1186/s12872-021-02117-z)
Supplement: Supplementary file 1 — Additional file 1: Table S1. Degree of linkage disequilibrium of MTHFR genetic polymorphisms between total CHD group and control group. [file 12872_2021_2117_MOESM1_ESM.docx]

| r^2^ | rs3737964 | rs2066470 | rs4846052 | rs1801133 | rs1801131 | rs1476413 | rs2274976 | rs4846048 | rs1889292 |
| --- | --- | --- | --- | --- | --- | --- | --- | --- | --- |
| rs535107 | 0.351 | 0.333 | 0.335 | 0.108 | 0.603 | 0.661 | 0.288 | 0.270 | 0.696 |
| rs3737964 | - | 0.010 | 0.004 | 0.054 | 0.246 | 0.292 | 0.012 | 0.642 | 0.342 |
| rs2066470 | - | - | 0.438 | 0.025 | 0.326 | 0.274 | 0.718 | 0.011 | 0.277 |
| rs4846052 | - | - | - | 0.043 | 0.288 | 0.246 | 0.435 | 0.001 | 0.295 |
| rs1801133 | - | - | - | - | 0.081 | 0.096 | 0.030 | 0.063 | 0.115 |
| rs1801131 | - | - | - | - | - | 0.592 | 0.296 | 0.208 | 0.598 |
| rs1476413 | - | - | - | - | - | - | 0.315 | 0.268 | 0.663 |
| rs2274976 | - | - | - | - | - | - | - | 0.015 | 0.306 |
| rs4846048 | - | - | - | - | - | - | - | - | 0.367 |

**Additional file 1: Table S1 Degree of linkage disequilibrium of MTHFR genetic polymorphisms between total CHD group and control group**

MTHFR=Methylenetetraphydrofolate reductase; CHD=congenital heart disease
