# Supplementary material for: Association analysis of maternal MTHFR gene polymorphisms and the occurrence of congenital heart disease in offspring
Source: BMC Cardiovasc Disord. 2021 Jun 14;21:298. doi: 10.1186/s12872-021-02117-z (PMC8204503; doi:10.1186/s12872-021-02117-z)
Supplement: Supplementary file 2 — Additional file 2: Table S2. Degree of linkage disequilibrium of MTHFR genetic polymorphisms between ASD group and control group. [file 12872_2021_2117_MOESM2_ESM.docx]

**Additional file 2: Table S2 Degree of linkage disequilibrium of MTHFR genetic polymorphisms between ASD group and control group**

MTHFR=Methylenetetraphydrofolate reductase; ASD=atrial septal defect

| r^2^ | rs3737964 | rs2066470 | rs4846052 | rs1801133 | rs1801131 | rs1476413 | rs2274976 | rs4846048 | rs1889292 |
| --- | --- | --- | --- | --- | --- | --- | --- | --- | --- |
| rs535107 | 0.288 | 0.306 | 0.238 | 0.079 | 0.592 | 0.626 | 0.275 | 0.258 | 0.667 |
| rs3737964 | - | 0.006 | 0.000 | 0.039 | 0.204 | 0.256 | 0.010 | 0.580 | 0.290 |
| rs2066470 | - | - | 0.357 | 0.018 | 0.389 | 0.287 | 0.652 | 0.007 | 0.315 |
| rs4846052 | - | - | - | 0.021 | 0.300 | 0.168 | 0.370 | 0.000 | 0.253 |
| rs1801133 | - | - | - | - | 0.075 | 0.086 | 0.020 | 0.056 | 0.083 |
| rs1801131 | - | - | - | - | - | 0.604 | 0.336 | 0.187 | 0.638 |
| rs1476413 | - | - | - | - | - | - | 0.309 | 0.249 | 0.629 |
| rs2274976 | - | - | - | - | - | - | - | 0.012 | 0.315 |
| rs4846048 | - | - | - | - | - | - | - | - | 0.317 |
