# Supplementary material for: Association analysis of maternal MTHFR gene polymorphisms and the occurrence of congenital heart disease in offspring
Source: BMC Cardiovasc Disord. 2021 Jun 14;21:298. doi: 10.1186/s12872-021-02117-z (PMC8204503; doi:10.1186/s12872-021-02117-z)
Supplement: Supplementary file 3 — Additional file 3: Table S3. Degree of linkage disequilibrium of MTHFR genetic polymorphisms between VSD group and control group. [file 12872_2021_2117_MOESM3_ESM.docx]

**Additional file 3: Table S3 Degree of linkage disequilibrium of MTHFR genetic polymorphisms between VSD group and control group**

MTHFR=Methylenetetraphydrofolate reductase; VSD=ventricular septal defect

| r^2^ | rs3737964 | rs2066470 | rs4846052 | rs1801133 | rs1801131 | rs1476413 | rs2274976 | rs4846048 | rs1889292 |
| --- | --- | --- | --- | --- | --- | --- | --- | --- | --- |
| rs535107 | 0.326 | 0.335 | 0.308 | 0.102 | 0.579 | 0.642 | 0.294 | 0.270 | 0.667 |
| rs3737964 | - | 0.007 | 0.000 | 0.049 | 0.221 | 0.266 | 0.010 | 0.647 | 0.315 |
| rs2066470 | - | - | 0.448 | 0.025 | 0.343 | 0.271 | 0.689 | 0.009 | 0.276 |
| rs4846052 | - | - | - | 0.047 | 0.289 | 0.219 | 0.441 | 0.000 | 0.259 |
| rs1801133 | - | - | - | - | 0.072 | 0.095 | 0.025 | 0.059 | 0.103 |
| rs1801131 | - | - | - | - | - | 0.560 | 0.302 | 0.181 | 0.573 |
| rs1476413 | - | - | - | - | - | - | 0.323 | 0.257 | 0.633 |
| rs2274976 | - | - | - | - | - | - | - | 0.013 | 0.315 |
| rs4846048 | - | - | - | - | - | - | - | - | 0.354 |
