# Supplementary material for: Association analysis of maternal MTHFR gene polymorphisms and the occurrence of congenital heart disease in offspring
Source: BMC Cardiovasc Disord. 2021 Jun 14;21:298. doi: 10.1186/s12872-021-02117-z (PMC8204503; doi:10.1186/s12872-021-02117-z)
Supplement: Supplementary file 4 — Additional file 4: Table S4. Degree of linkage disequilibrium of MTHFR genetic polymorphisms between PDA group and control group. [file 12872_2021_2117_MOESM4_ESM.docx]

**Additional file 4: Table S4 Degree of linkage disequilibrium of MTHFR genetic polymorphisms between PDA group and control group**

MTHFR=Methylenetetraphydrofolate reductase; PDA=patent ductus arteriosus

| r^2^ | rs3737964 | rs2066470 | rs4846052 | rs1801133 | rs1801131 | rs1476413 | rs2274976 | rs4846048 | rs1889292 |
| --- | --- | --- | --- | --- | --- | --- | --- | --- | --- |
| rs535107 | 0.346 | 0.284 | 0.274 | 0.087 | 0.578 | 0.654 | 0.262 | 0.277 | 0.691 |
| rs3737964 | - | 0.008 | 0.007 | 0.049 | 0.232 | 0.289 | 0.011 | 0.598 | 0.336 |
| rs2066470 | - | - | 0.334 | 0.017 | 0.318 | 0.257 | 0.681 | 0.010 | 0.285 |
| rs4846052 | - | - | - | 0.042 | 0.275 | 0.196 | 0.338 | 0.002 | 0.267 |
| rs1801133 | - | - | - | - | 0.082 | 0.088 | 0.020 | 0.062 | 0.094 |
| rs1801131 | - | - | - | - | - | 0.579 | 0.279 | 0.210 | 0.600 |
| rs1476413 | - | - | - | - | - | - | 0.280 | 0.260 | 0.625 |
| rs2274976 | - | - | - | - | - | - | - | 0.014 | 0.289 |
| rs4846048 | - | - | - | - | - | - | - | - | 0.351 |
